# Bortezomib in previously treated phosphatase and tension homology‐deficient patients with advanced intrahepatic cholangiocarcinoma: An open‐label, prospective and single‐centre phase II trial

**DOI:** 10.1002/ctm2.1675

**Published:** 2024-04-30

**Authors:** Tian‐mei Zeng, Tian‐yi Jiang, Guang Yang, Zhuo Cheng, Cheng Lou, Wei Wei, Chen‐jie Tao, Shouzi Hu, Hui Wang, Xiao‐wen Cui, Ye‐xiong Tan, Li‐wei Dong, Hong‐yang Wang, Zhen‐gang Yuan

**Affiliations:** ^1^ Department of Oncology Eastern Hepatobiliary Surgery Hospital, The Naval Medical University Shanghai China; ^2^ National Center for Liver Cancer, The Naval Medical University Shanghai China; ^3^ Department of Hepatobiliary Diseases Eastern Hepatobiliary Surgery Hospital, The Naval Medical University Shanghai China

**Keywords:** bortezomib, intrahepatic cholangiocarcinoma, PTEN

## Abstract

**Introduction:**

Intrahepatic cholangiocarcinoma (ICC) is characterized by a dismal prognosis with limited therapeutic alternatives. To explore phosphatase and tension homolog (PTEN) as a biomarker for proteasome inhibition in ICC, we conducted a phase II trial to assess the second‐line efficacy of bortezomib in PTEN‐deficient advanced ICC patients.

**Methods:**

A total of 130 patients with advanced ICC in our centre were screened by PTEN immunohistochemical staining between 1 July 2017, and 31 December 2021, and 16 patients were ultimately enrolled and treated with single‐agent bortezomib 1.3 mg/m^2^ on days 1, 4, 8 and 11 of a 21‐day cycle. The primary endpoint was the objective response rate (ORR) according to Response Evaluation Criteria in Solid Tumors v1.1.

**Results:**

The median follow‐up was 6.55 months (95% confidence interval [CI]: 0.7–19.9 months). Among the 16 enrolled patients, the ORR was 18.75% (3/16) and the disease control rate was 43.75% (7/16). The median progress‐free survival was 2.95 months (95% CI: 2.1–5.1 months) and the median overall survival (mOS) was 7.2 months (95% CI: 0.7–21.6 months) in the intent‐to‐treat‐patients. Treatment‐related adverse events of any grade were reported in 16 patients, with thrombopenia being the most common toxicity. Patients with PTEN staining scores of 0 were more likely to benefit from bortezomib than those with staining scores > 0.

**Conclusions:**

Bortezomib yielded an encouraging objective response and a favourable OS as a second‐line agent in PTEN‐deficient ICC patients. Our findings suggest bortezomib as a promising therapeutic option for patients with PTEN‐deficient ICC.

**Highlights:**

- There is a limited strategy for the second‐line option of intrahepatic cholangiocarcinoma (ICC).
- This investigator‐initiated phase 2 study evaluated bortezomib in ICC patients with phosphatase and tension homology deficiency.
- The overall response rate was 18.75% and the overall survival was 7.2 months in the intent‐to‐treat cohort.
- These results justify further developing bortezomib in ICC patients with PTEN deficiency.

## Graphical abstract

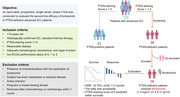

## 1 INTRODUCTION

Intrahepatic cholangiocarcinoma (ICC) is the second most frequent hepatic malignancy accounting for approximately 10% of primary liver cancers.^1^ ICC, a subclass of biliary tract cancers (BTCs), exhibits an aggressive phenotype and dismal prognosis. Checkpoint inhibitors combined with chemotherapy have emerged as the new standard treatment in the palliative setting.^2^ Regarding second‐line treatment options, the ABC‐06 trial stands as the sole prospective randomized phase III study evaluating the efficacy of chemotherapy combined with active supportive care in BTC patients, among whom 44% were diagnosed with ICC. However, the addition of FOLFOX in the ABC‐06 trial only demonstrated a marginal improvement in median overall survival (mOS) to 6.2 months, with an inadequate objective response rate of 5%.^3^ Recent advancements in understanding the molecular pathogenesis of BTCs and the identification of relevant biomarkers have facilitated the development of targeted therapies. Patients harbouring rare genetic alterations, such as FGFR2 fusions, IDH1/2 mutations, and microsatellite instability‐high, now have more therapeutic options following the failure of first‐line therapy.^4^

Phosphatase and tension homolog (PTEN) stands out as one of the most frequently mutated genes in human malignancies.^5^ PTEN deficiency has been identified as a predictor of poor prognosis in cholangiocarcinoma.^6^ Our previous study revealed that PTEN deficiency promotes protein synthesis, proteasome subunit expression, and proteolytic activity, establishing a reliance on the proteasome for ICC cell growth and survival.^7^ Consistently, our previous study revealed that PTEN deficiency occurs in 30% of patients with advanced ICC^8^ and down‐regulation of PTEN occurs in 60% of patients with advanced gallbladder cancer (GBC).^9^ While PTEN deficiency commonly signifies worse outcomes and increases metastases in BTC,^10^ it also exposes a vulnerability to proteasome inhibition.^7^, ^9^ Mechanistically, PTEN deficiency promotes proteasome subunit expression, and proteolytic activity by inhibiting the ARE‐related transcriptional suppressors BACH1 and MAFF, thereby fostering dependence on the proteasome for ICC cell growth and survival.^7^ These findings suggest that targeting the proteasomes could offer a promising therapeutic avenue for ICC patients with PTEN deficiency. However, a phase II trial of bortezomib, a selective inhibitor of the 26S proteasome, in unselected BTC patients failed to meet its primary endpoints years ago, despite some cases displaying a response to bortezomib.^11^ To date, no study has validated the efficacy of bortezomib in PTEN‐deficient ICC patients. Thus, we conducted this trial to evaluate the clinical efficacy and safety of bortezomib in a selected cohort of PTEN‐deficient ICC patients.

## 2 METHODS

### 2.1 Patients

Eligible patients screened at Eastern Hepatobiliary Surgery Hospital between 1 July 2017 and 31 December 2021, had histological evidence of unresectable adenocarcinoma only of the intrahepatic bile ducts. Central confirmation of the negative or low expression of PTEN was required. Other inclusion criteria included age older than 18 years, measurable disease, adequate hematologic functions (white blood cells > 3.0 × 10^9^/L, platelets > 100 × 10^9^/L and haemoglobin≥ 85 g/L) and normal biochemical functions (bilirubin ≤ 1.5 × institutional upper limit of normal and creatinine≤ 1.5 × institutional upper limit of normal or glomerular filtration rate ≥60 mL/min/1.73m^2^), standard first‐line therapy (gemcitabine combined with cisplatin or gemcitabine combined with oxaliplatin if cisplatin is not indicated), and Eastern Cooperative Oncology Group (ECOG) performance status as low as 0, 1 or 2.

### 2.2 Trial design and treatment

In this open‐label, single‐arm and phase II trial, bortezomib (Hauser) was administered at a dose of 1.3 mg/m^2^ on days 1, 4, 8 and 11 of a 21‐day cycle within one month after signing informed consent. The single drug was subcutaneously injected to alleviate potential peripheral neuritis. Treatment was discontinued when patients suffered from unacceptable adverse events or disease progression. Toxicity was graded according to the National Cancer Institute‐Common Toxicity Criteria, version 5.0. Dose modifications were made for ≥ grade 3 toxicity (except neuropathy). Patients who experienced treatment‐related neuropathic pain and/or peripheral neuropathy were managed according to established dose‐modification guidelines.^12^ Dose reduction levels were 1 and 0.7 mg/m^2^ per dose. Patients with a requirement of more than two dose reductions were removed from the study. Our study was approved by the Ethics Committee of the Eastern Hepatobiliary Surgery Hospital (EHBHKY2017‐01‐002) and registered on ClinicalTrials.gov (NCT03345303). The trial was performed in accordance with the Declaration of Helsinki. All patients signed written informed consent before enrollment. Bortezomib was provided for free. The data cutoff date for the final analysis was 31 December 2022.

### 2.3 Outcome assessment

Therapeutic efficacy including computed tomography scans of the chest and abdomen and magnetic resonance imaging of the liver were conducted every two cycles. Blood routine, liver and kidney function, electrolytes and tumour markers (including serum CEA and CA199 levels) were tested at the beginning of each cycle. The primary efficacy endpoint was the objective response rate (ORR) based on investigator assessments, according to the Response Evaluation Criteria in Solid Tumors (RECIST version 1.1). Secondary endpoints included disease control rate (DCR), progression‐free survival (PFS), and OS. An objective response was defined as a complete response or a partial response (PR). Disease control was defined as complete response, PR, or stable disease. PFS was defined as the time from the initiation of therapy to the date of progressive disease. OS was defined as the time from the initiation of therapy to death or last follow‐up.

### 2.4 Immunohistochemistry staining for PTEN

Immunohistochemistry (IHC) was performed as previously described.^10^ Briefly, the tissues were fixed in 10% formalin overnight and embedded in paraffin. Endogenous peroxidases were inactivated using 3% hydrogen peroxide. Nonspecific signals were blocked using 1% bovine serum albumin. The tumour samples were stained with the following primary antibodies: PTEN (CST, 9559, 1:50) and CK19 (Proteintech, 10712‐1‐AP, 1:500). After overnight incubation, the slides were washed and incubated with the secondary antibody (Horseradish peroxidase polymer; Biocare Medical) for 30 min at room temperature. The slides were washed three times and stained with 3, 3′‐diaminobenzidine substrate (Thermo Fisher Scientific). Then the slides were counterstained with hematoxylin and mounted with a mounting medium. The human tissue sections were blindly reviewed and scored for staining scores (0–3) in tumour cells by two independent pathologists using the Aperio Image Scope Viewer. If the pathologists disagreed with the staining scores of PTEN, a third pathologist was invited to give the final assessment. The intensity was scored as follows: 0, negative; 1, weak; 2, moderate; and 3, strong. The frequency of positive cells was defined as follows: 0, less than 5%; 0.25, 5%–25%; 0.5, 26%–50%; 0.75, 51%–75%; and 1, greater than 75%. When the staining was heterogeneous, we scored it as follows: each component was independently scored and summed for the results. For example, a specimen containing 75% tumour cells with moderate intensity (2 × 0.75 = 1.5) and another 25% tumour cells with weak intensity (1 × 0.25 = 0.25) received a final score of 1.5 + 0.25 = 1.75. Patients with PTEN IHC scores ≤ 1.5 were enrolled in this clinical trial. For statistical analyses, score 0 was considered as negative, and 0 < scores ≤ 1.5 as low expression.

### 2.5 In situ hybridization for PTEN mRNA (RNA Scope technology)

Chromogenic RNA Scope for PTEN mRNA was performed on sections from paraffin‐embedded tumour tissues following a previously described standard protocol.^13^ Briefly, sections were cut at 5 µm and air‐dried before pretreatments. For all probes, the tumour tissues were subjected to a standard pretreatment protocol. Three RNA Scope probes from advanced cell diagnostics (ACD) were used in this study: Hs‐PTEN (cat.408511), positive control probe Homo sapiens PPIB (Hs‐PPIB, cat.313901), and negative control probe dihydrodipicolinate reductase (bacterial DapB, cat.310043). Detection of specific probe binding sites was performed with RNA Scope 2.5 HD Reagent Kit (Brown) from ACD (cat.322300). For semiquantitative microscopic evaluations of PTEN mRNA detection by RNA Scope, a 4‐tier scoring system was developed: ‐, no spots in tumour cells; +, a few spots in most cells, ++, a moderate number of spots in all cells and +++, a high number of spots in all cells.

### 2.6 Targeted next‐generation sequencing for PTEN

We performed targeted next‐generation sequencing (NGS) for the complete genomic sequence of the *PTEN* gene. The target size was 108 kb of captured DNA after the removal of repetitive sequences. Genomic capture from the pooled libraries was performed by a TargetSeq Enrichment Kit (iGeneTech). The libraries were sequenced on an Illumina HiSeq X Ten platform, generating 2 × 150 bp paired‐end reads. The sequenced reads were aligned to the NCBI human reference genome GRCh37 (hg19) using the Burrows‐Wheeler Aligner 30. Variant detection was conducted for Indels and single nucleotide variants (SNVs) using a pipeline based on the Genome Analyzer Toolkit (GATK). Copy number variations (CNVs) were called by using CNVseq. Briefly, base qualities were recalibrated, sequence reads around Indels were realigned, and the GATK Unified Genotyper was subsequently employed to perform consensus calling and to identify SNVs. Only well‐mapped reads (mapping quality of ≥ 30 and number of mismatches within a 40‐bp window of ≤ 3) were used as input for the Genotyper.

### 2.7 Statistical analysis

Continuous data with normal distributions were presented as means and standard deviations and those with screwed distributions were presented as medians and ranges. Categorical data were presented as frequencies or percentages. Survival curves were drawn using the Kaplan‐Meier method. *p*‐Values < .05 were considered statistically significant. The 95% confidence intervals (95% CIs) were calculated using the exact Clopper‐Pearson method. All statistical analyses were performed using the SPSS 21.0 (IBM) and GraphPad (V8.0).

## 3 RESULTS

### 3.1 Patients

**FIGURE 1:**
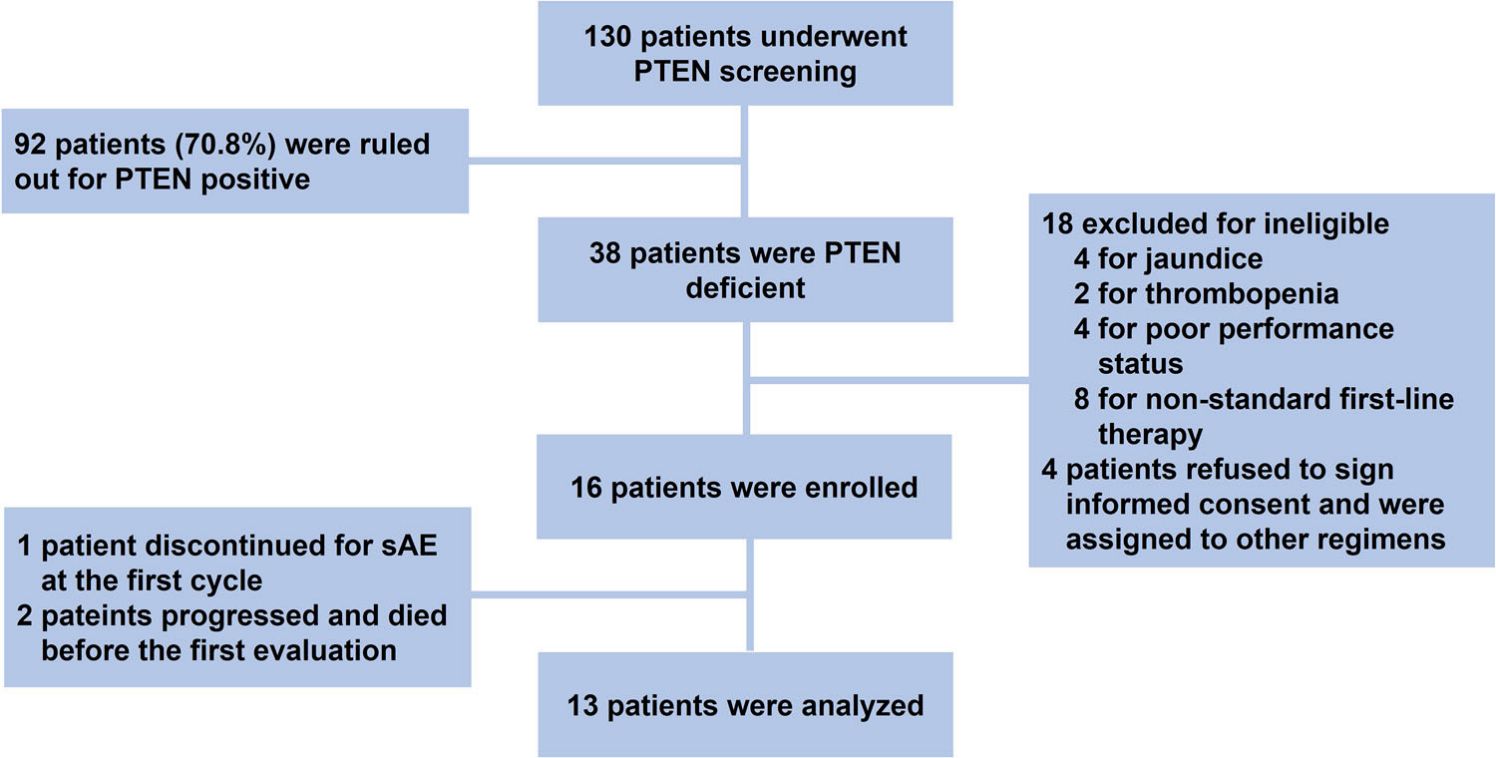
Flow chart of patient inclusion. sAE: serious adverse event.

A total of 130 ICC patients were screened at our centre from 1 July 2017 to 31 December 2021, for PTEN expression (Figure 1). Among them, 92 patients (71%) exhibited positive PTEN expression (staining scores > 1.5) and were excluded, while 38 patients (29%) were verified as having PTEN deficiency (PTEN staining scores of 0–1.5). Subsequently, ten patients were deemed ineligible due to jaundice, thrombopenia, or poor performance status, and eight patients did not receive gemcitabine based chemotherapy as first‐line regimen. Additionally, four patients declined to provide informed consent. Ultimately, a total of 16 patients were enrolled and received bortezomib as their second‐line therapy. The clinical characteristics of the patients are shown in Table 1. The median age was 64 years, ranging from 30 to 72 years old, with 5 (31%) female patients. The majority of patients had moderately differentiated tumours (11, 69%) and all had metastasis to at least one distant organ.

**TABLE 1:** Patient baseline clinical characteristics.

Patients (*n*)	16
Age (years), median (range)	64 (30–72)
Female, *n* (%)	5 (31.25)
ECOG performance status, *n* (%)	
0	3 (18.75)
1	11 (68.75)
2	2 (12.5)
Grade of differentiation	
Moderately	11 (68.75)
Poorly	5 (31.25)
Tumor status	
Local advanced	0
Metastatic	16 (100)
Numbers of metastasis organs, *n* (%)	
1	6 (37.5)
2	6 (37.5)
3−4	4 (25)
Carbohydrate antigen 19‐9 ≥ upper limit of normal, *n* (%)	14 (87.5)
Hepatitis B, *n* (%)	
HBsAg positive	6 (37.5)
HBV‐DNA <50 IU/mL	6 (37.5)
HBV‐DNA >50 IU/mL	0
HBsAg negative	10 (62.5)
Prior treatment	
GC	14 (87.5)
GEMOX	2 (12.5)
PTEN‐deficient expression	
Negative	5 (31.25)
Low expression	11 (68.75)

Abbreviations: ECOG, Eastern Cooperative Oncology Group; GC, gemcitabine in combination with cisplatin; GEMOX, gemcitabine in combination with oxaliplatin; HBV, hepatitis B virus; PTEN, phosphatase and tension homolog.

### 3.2 Tumour response and PTEN expression

The ORR was 18.75% (95% CI:4%–45.6%) and the DCR was 43.75% (95% CI: 19.8%–70.1%) in the intent‐to‐treat (ITT) cohort. It was worth noting that three patients did not receive an assessment for the efficacy of bortezomib. One patient experienced severe peripheral neuritis after the third dose of bortezomib and withdrew from the study to receive fluorouracil‐based chemotherapy. This patient experienced rapid disease progression and had a short survival time of less than 4 months. The other two patients both suffered from tumour‐related biliary tract obstruction before the second cycle and succumbed to liver function failure within a month. Consequently, the ORR and DCR in the per‐protocol (PP) cohorts were 23.08% (95%CI: 5%–53.8%) and 53.85% (95%CI: 25.1%–80.8%), respectively.

**FIGURE 2:**
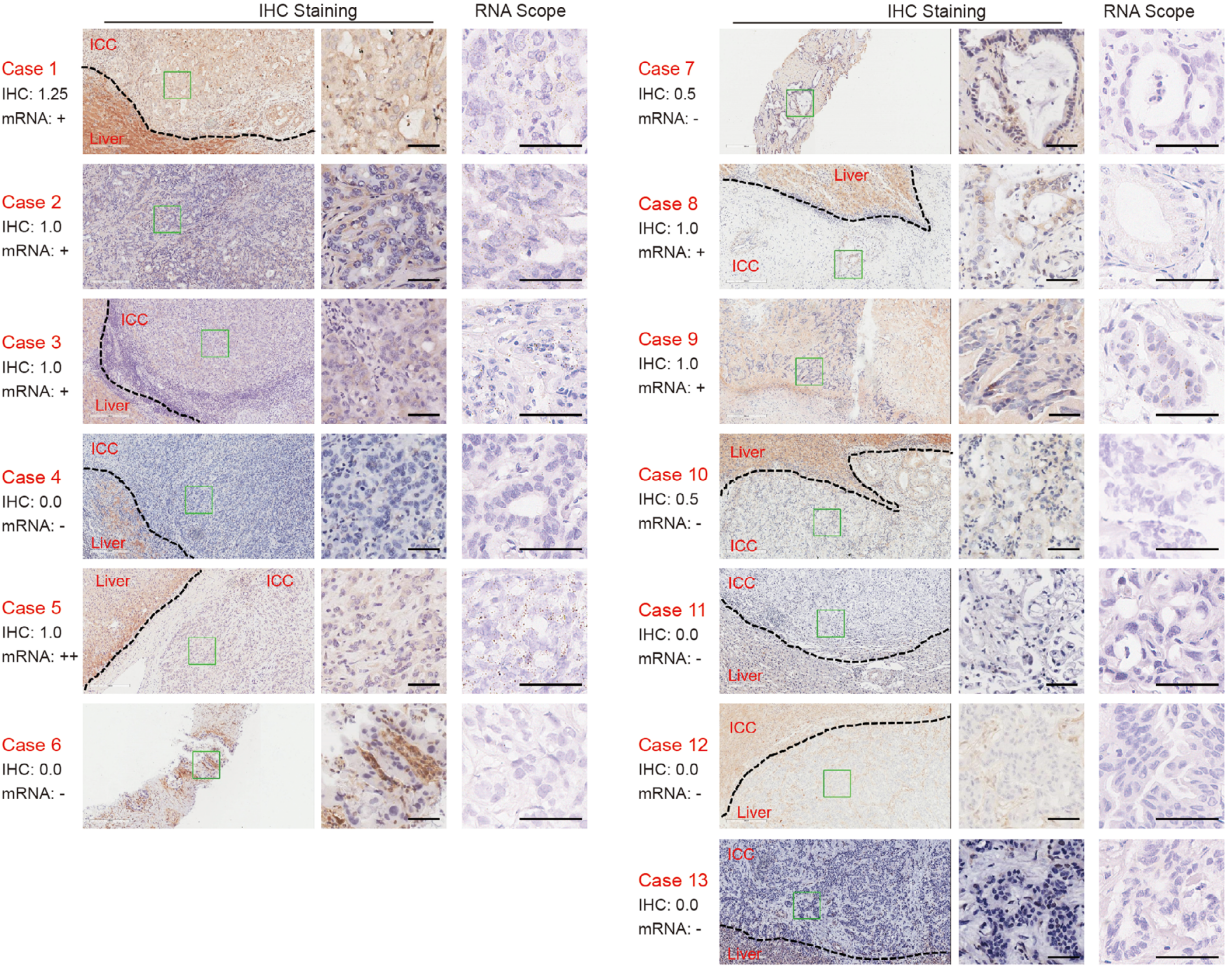
The immunohistochemistry (IHC) and RNA scope images of the per‐protocol cohort.

**FIGURE 3:**
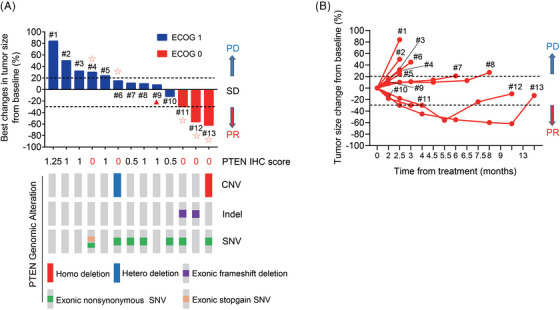
Radiographic response in phosphatase and tension homolog (PTEN)‐deficient intrahepatic cholangiocarcinoma (ICC) patients treated with bortezomib. (A) Waterfall plot showing the best change in tumour size from baseline. *PTEN* genomic alterations for each patient. (B) Spider plot showing depth and duration of tumour regressions. 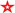: patients with PTEN negative. 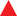: patients with new lesions.

In the PP cohort, PTEN protein and mRNA expression levels were determined for each patient using IHC and RNA Scope staining (Figure 2). Additionally, targeted NGS for the *PTEN* gene was performed on these specimens, and the protein/mRNA expression and *PTEN* genomic alterations for each patient are depicted in Table 2. The best reductions per patient, along with their PTEN testing results, are illustrated in Figure 3A. The changes in measurable lesions over time are displayed in Figure 3B. These results revealed a perfect alignment between PTEN staining score 0 and mRNA negativity, as well as DNA functional deletion. Notably, the three patients with remarkable tumour shrinkage (11#, 12# and 13# in Figure 3A) were all found to be PTEN negative, suggesting that PTEN negativity might serve as a biomarker for predicting the response to bortezomib.

**TABLE 2:** Individual patient data including immunohistochemistry (IHC) score, genomic alteration, response to bortezomib and dosage reduction.

Case	PTEN			Genomic alteration		Response to bortezomib	Dosage reduction					
	Score	RNA	CNV	Indel	SNP		Cycle 1	Cycle 2	Cycle 3	Cycle 4	Cycle 5	Cycle 6
1	1.25	+	NA		Synonymous SNV	PD	1.3 mg/m^2^	1.0 mg/m^2^	PD			
2	1	+	NA		Nonsynonymous SNV	PD	1.3 mg/m^2^	1.0 mg/m^2^	PD			
3	1	+	NA		NA	PD	1.3 mg/m^2^	1.0 mg/m^2^	PD			
4	0	–	Hetero deletion		Nonsynonymous SNV	PD	1.3 mg/m^2^	1.0 mg/m^2^	1.0 mg/m^2^	0.7 mg/m^2^	PD	
5	1	++	NA		NA	PD	1.3 mg/m^2^	1.0 mg/m^2^	PD			
6	0	–	NA		Nonsynonymous SNV	SD+	1.3 mg/m^2^	1.0 mg/m^2^	1.0 mg/m^2^	1.0 mg/m^2^	PD	
7	0.5	–	NA		NA	SD+	1.3 mg/m^2^	1.0 mg/m^2^	0.7 mg/m^2^	0.7 mg/m^2^	PD	
8	1	+	NA		Nonsynonymous SNV	SD+	1.3 mg/m^2^	1.3 mg/m^2^	1.0 mg/m^2^	1.0 mg/m^2^	0.7 mg/m^2^	……to PD
9	1	–	NA		NA	SD+	1.3 mg/m^2^	1.0 mg/m^2^	PD			
10	0.5	–	NA		Nonsynonymous SNV	SD‐	1.3 mg/m^2^	1.0 mg/m^2^	0.7 mg/m^2^	Discontinued^a^		PD
11	0	–	NA	Frameshift deletion	Nonsynonymous SNV	PR	1.3 mg/m^2^	1.0 mg/m^2^	1.0 mg/m^2^	1.0 mg/m^2^	0.7 mg/m^2^	……to PD
12	0	–	NA	Frameshift deletion		PR	1.3 mg/m^2^	1.3 mg/m^2^	1.0 mg/m^2^	……to PD		
13	0	–	Homo deletion		Nonsynonymous SNV	PR	1.3 mg/m^2^	1.3 mg/m^2^	1.3 mg/m^2^	1.0 mg/m^2^	1.0 mg/m^2^	……to PD

^a^ The patient acquired a disease stable after 2 cycles but suspended treatment for personal reasons.

Abbreviations: CNV, copy number variation; NA, not available; PD, progression disease; PR, partial response; PTEN, phosphatase and tension homolog; SD‐, reduction stable disease; SD+, enlargement stable disease; SNP, single nucleotide polymorphism.

### 3.3 Patient survival

**FIGURE 4:**
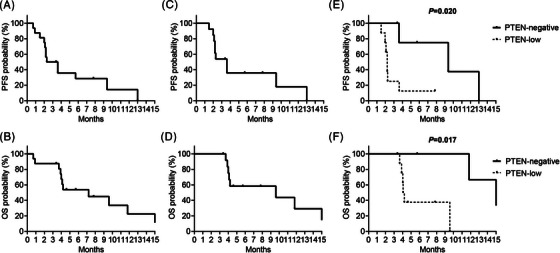
Kaplan‐Meier estimates of survival outcomes in phosphatase and tension homolog (PTEN)‐deficient intrahepatic cholangiocarcinoma (ICC) patients treated with bortezomib. (A) The progression‐free survival (PFS) of the intent‐to‐treat (ITT) cohort (2.95 months [95% confidence interval [CI]: 2.1–5.1 months]). (B) The overall survival (OS) of the ITT cohort (7.2 months [95% CI: 0.7–21.6 months]). (C) The PFS of the per‐protocol (PP) cohort (3.6 months [95% CI: 2.1–5.1 months]). (D) The OS of the PP cohort (9.6 months [95% CI: 0.7–21.6 months]). (E) The PFS in patients with different PTEN staining scores. The PFS of PTEN‐negative (score 0) and PTEN low expression (score > 0) were 9.4 and 2.2 months (*p* = .020) (F) The OS in patients with different PTEN staining scores. The OS of PTEN‐negative (score 0) and PTEN low expression (score > 0) were 15.2 and 4.1 months (*p* = .017).

At data cutoff, among the ITT group, 15 patients experienced progressive disease, and twelve patients had died. The median follow‐up was 6.55 months (0.7–17.2 months). In the ITT cohort, the median PFS was 2.95 months (95% CI: 2.1‐5.1 months) and the median OS was 7.2 months (95% CI: 0.7–21.6 months) (Figure 4A,B). In the PP analysis, the median PFS was 3.6 months (95% CI: 2.1–5.1 months) and the median OS were 9.6 months (95% CI: 0.7‐21.6 months) (Figure 4C,D). Among the patients who achieved a PR, one patient exhibited extensive tumour regression in the lung, with a PFS of 13 months.

### 3.4 Exploratory analysis

While the moderate sample size constrained our ability to explore potential biomarkers fully, we observed that patients with good performance status exhibited significantly higher response rates compared to those with poorer conditions (*p *= .003). Notably, all patients who achieved PR (3/16, 18.8%) had good performance status (Figure 3A), whereas the two patients with an ECOG score of 2 experienced rapid disease progression and died before the first evaluation. Furthermore, patients with a PTEN staining score of 0 (Figure 3A) were more likely to benefit from bortezomib than those with a staining score above 0 (*p *= .04). Three out of four patients with tumour regression were confirmed PTEN negative (Figure 3A). In contrast, seven of the nine patients (77.8%) with varying increases in tumour size had weakly positive PTEN staining (Figure 3A). The PFS and OS of PTEN‐negative (score 0) patients were 9.4 and 15.2 months, respectively, compared with 2.2 and 4.1 months, respectively, for patients with low PTEN (score > 0). These findings suggested that PTEN‐negative status, indicated by a staining score of 0, predicted a better prognosis in ICC patients treated with bortezomib (*p* = .020 and .017, respectively) (Figure 4E,F). Additionally, other characteristics such as hepatitis B virus infection status and prior treatment regimen didn't show any correlations with treatment efficacy (*p *= .518 and 1.000, respectively).

### 3.5 Safety

Toxicity was evaluated among the 16 patients who received at least one dose of bortezomib (Table 3). Treatment‐related adverse events were reported in 16 (100%) patients, although no treatment‐related deaths were reported in this cohort. Platelet count decrease was the most common toxicity, which was observed in 14 (88%) patients of any grade and 9 (56%) of grades 3–4. Peripheral sensory neuropathy was reported in ten (63%) patients, one of whom discontinued treatment due to severe pain. Other reported treatment‐related toxicities included diarrhoea, abdominal pain, nausea, fatigue, impaired liver function, fever, neutrophil count decreased, anaemia and gastric haemorrhage. Despite necessitating dose reductions in all patients (Table 2), the safety profile of bortezomib in ICC patients was deemed acceptable in this study.

**TABLE 3:** Summary of drug‐related adverse events in all 16 patients on trial.

Event in 16 patients	Any grade, *n* (%)	Grades 3–4, *n* (%)
Platelet count decrease	14 (87.5)	9 (56.25)
Peripheral sensory neuropathy	10 (62.5)	2 (12.5)
Diarrhoea	6 (37.5)	1 (6.25)
Abdominal pain	5 (31.25)	0
Nausea	4 (25)	0
Fatigue	3 (18.75)	0
Alanine aminotransferase increase	3 (18.75)	1 (6.25)
Blood bilirubin increased	3 (18.75)	1 (6.25)
Fever	3 (18.75)	2 (12.5)
Neutrophil count decreased	3 (18.75)	1 (6.25)
Anaemia	2 (12.5)	0
Gastric haemorrhage	1 (6.25)	0

## 4 DISCUSSION

Precise treatment of tumours relies on accurate stratification of their molecular typing, which facilitates the identification of therapeutic targets and prognosis. To the best of our knowledge, there are no clinical trials exploiting the association between therapeutic efficacy and PTEN deficiency in cholangiocarcinoma, despite the early recognition of the predictive potential of PTEN deficiency.^6^ Our previous research revealed that PTEN deficiency increased the proteolytic activity in ICC.^7^ Consequently, we initiated this prospective clinical trial, unprecedentedly evaluating the proteasome inhibitor bortezomib in a selected ICC cohort with PTEN deficiency. Notably, a prior phase II trial assessing bortezomib's therapeutic efficacy in unselected BTC patients failed to meet its primary endpoint. However, it's noteworthy that one ICC patient with thoracic lymph node metastases in that trial achieved an unconfirmed PR, supporting the potential application of proteasome inhibitors in a subgroup of ICC patients.

ICC is an invasive adenocarcinoma with a poor prognosis and limited benefit from subsequent therapies. Our centre's study, consistent with prior research,^6^ demonstrated that PTEN deficiency further aggravates the poor prognosis of ICC patients.^10^ Significantly, many PTEN‐deficient patients in our study experienced aggressive disease progression or poor performance status post‐standard therapy, precluding further treatment options. Among the 16 enrolled PTEN‐deficient patients, thirteen were in relatively worse condition at disease progression (ECOG scores of 1 and 2) compared to participants in other second‐line studies.^3^, ^14^, ^15^ Moreover, 62.5% of patients exhibited metastasis to more than one organ, indicative of a substantial tumour burden. These poor clinical characteristics associated with PTEN deficiency suggest a more aggressive biological phenotype.

To date, no drugs and regimens other than FGFR inhibitors have succeeded in significantly prolonging PFS or OS following GC treatment. However, FGFR inhibitors only benefit less than 15% of ICC patients,^16^ with an even lower rate (6.14%) in the Chinese population.^17^ In contrast, approximately 30% of ICC patients in our study exhibited PTEN deficiency. Notably, the ORR in our study was 23.08% in the PP cohort, which was only inferior to previous reports on FGFR inhibitors,^15^, ^18^ yet indicated a significant improvement compared to other second‐line regimens, such as FOLFOX (5%),^3^ regorafenib (11%),^19^ and lenvatinib plus pembrolizumab (10%).^20^ Additionally, the DCR was 53.85% in the PP cohort, exceeding the reported 52.63% in unselected BTC patients (also in the PP cohort).^11^

Even though stratification by PTEN deficiency was not the first or the unique target in precise treatment for ICC, PTEN was undoubtedly a convenient single genetic biomarker at the protein level which is different from other targets such as *FGFR2* fusions or *IDH1* mutations. Screening patients harbouring *FGFR2* fusions or *IDH1* mutations requires DNA sequencing, which is expensive and time‐consuming. By comparing the detecting methods adopted in this study, including IHC, NGS and RNA scope technology, the results showed a solid correlation between PTEN protein expression and genetic alterations. Consistently, in our previous study, we also performed IHC and gene sequencing of PTEN in 50 ICC patients, which also showed that PTEN deficiency is significantly related to genetic alterations.^8^ Generally, when PTEN homozygous deletion or frameshift deletion occurs, the protein expression of PTEN is negative. Therefore, we recommended IHC testing for PTEN at the beginning of screening. Certainly, both IHC and NGS testing of PTEN should be performed if the samples are sufficient.

ICC patients who receive GC in the standard care setting have a short life expectancy of approximately 3.7 months without subsequent therapies,^21^ let alone those with PTEN deficiency. However, in our study, patients who received at least two cycles of bortezomib treatment reached a median PFS of 3.6 months and an mOS of 9.6 months. These results are comparable to the positive outcomes reported in several clinical trials, including ClarIDHy, ABC06 and the BTC cohort of the LEAP 005 study. Our findings indicated that in patients with PTEN loss or *PTEN* loss‐of‐function mutations, the use of this single agent with acceptable toxicities may prolong patient survival.

Recently, the first randomized clinical trial evaluating anti‐programmed cell death‐Ligand 1 in combination with GC as initial therapy for advanced BTC patients reached its primary endpoint, leading immune checkpoint inhibitors (ICIs) to the standard treatment for BTC patients.^2^ However, patients with *PTEN* mutations or losses at the protein level exhibited intrinsic resistance to ICI therapy in multiple tumours.^16^, ^22^, ^23^, ^24^ Our research provides a novel therapeutic option for this particular subgroup of ICC patients with aggressive tumour biological behaviours that make it difficult to benefit from ICI therapy.

Toxicities associated with subcutaneous injections of bortezomib were deemed acceptable. However, dose reductions were required in all patients, with a significantly higher incidence than in other clinical trials. In those trials, the combination of bortezomib with glucocorticoids may have contributed to neuropathy and elevated platelets.^25^, ^26^ The safety of glucocorticoids in patients with solid tumours still warrants further investigation.

One major limitation of this study is the small sample size, which is due to the strict inclusion criteria for the target population. Only 12% of the screened population were enrolled. Approximately 71% of patients were excluded due to positive PTEN expression, while 8% were excluded due to poor performance status and insufficient organ function. The eight patients with PTEN deficiency excluded from non‐standard first‐line treatment were all because of poor conditions at the time of diagnosis, restraining them from the use of combined chemotherapy, which further supports the association between PTEN deficiency and poor prognosis. Although the small sample size limited further biomarker analysis, it is noteworthy that the response could be further stratified based on performance status and PTEN expression levels in our study. Future studies are needed to explore other potential biomarkers predicting response to bortezomib in larger cohorts. Preclinical data suggest that bortezomib enhances the activity of gemcitabine.^27^ In conjunction with our findings, a larger‐scale study is currently underway at our centre to evaluate the efficacy of bortezomib in combination with GC as first‐line therapy (ChiCTR2000035916). This phase 2, randomized clinical trial enrolled not only ICC patients but also patients with GBC as our further research showed PTEN deficiency could be tested in about 60% of patients with GBC and also facilitate the therapeutic vulnerability to bortezomib.^9^ In this ongoing trial, PTEN status is a critical stratification factor that could further validate the relationship between PTEN deficiency and bortezomib sensitivity in BTC patients.

## 5 CONCLUSION

Bortezomib displayed encouraging efficacy with manageable toxicities as a second‐line therapy for patients with PTEN‐deficient ICC, significantly prolonging OS to a median of 9.6 months in the PP cohort. Our findings open the possibility for a chemotherapy‐free therapeutic option for selected ICC populations with PTEN deficiency.

## AUTHOR CONTRIBUTIONS

Hong‐yang Wang, Li‐wei Dong and Zhen‐gang Yuan designed this study and take responsibility for the integrity of the data and the accuracy of the data analysis. Data collection: Tian‐mei Zeng, Guang Yang, Cheng Lou, Wei Wei, Chen‐jie Tao and Shou‐zi Hu; Data analysis: Tian‐mei Zeng and Zhuo Cheng; Morphological performance and pathological analysis: Tian‐Yi Jiang, Xiao‐Wen Cui, Li‐Wei Dong and Ye‐xiong Tan; Drafting and revision of the manuscript: Tian‐mei Zeng, Li‐Wei Dong and Tian‐Yi Jiang

## CONFLICT OF INTEREST STATEMENT

The authors declare no conflict of interest.

## FUNDING INFORMATION

This work was supported by the Clinical Research Plan of Shanghai Hospital Development Center (SHDC12019 × 04 and SHDC2020CR2011A), the National Natural Science Foundation of China (32270814, 81988101, 82172895 and 91859205), the key technologies Research and Development Program of China (2022YFC2503704) and Shanghai Sailing Program (21YF1457900).

## ETHICS STATEMENT

The study was approved by the Ethics Committee of the Eastern Hepatobiliary Surgery Hospital (EHBHKY2017‐01‐002). All patients signed written informed consent before enrollment.

## CLINICAL TRIAL REGISTRATION

ClinicalTrials.gov (NCT03345303).

## Data Availability

The genomic raw sequencing data generated in this study have been deposited in the Genome Sequence Archive in the National Genomics Data Center China National Center for Bioinformation(https://ngdc.cncb.ac.cn/gsa‐human/). The accession code is HRA003603. The clinical individual participant data will not be shared.

## References

[1] Lo EC , Rucker AN , Federle MP . Hepatocellular carcinoma and intrahepatic cholangiocarcinoma: imaging for diagnosis, tumor response to treatment and liver response to radiation. Semin Radiat Oncol. 2018;28:267‐276. 30309637 10.1016/j.semradonc.2018.06.010

[2] Oh D‐Y , He AR , Qin S , et al. A phase 3 randomized, double‐blind, placebo‐controlled study of durvalumab in combination with gemcitabine plus cisplatin (GemCis) in patients (pts) with advanced biliary tract cancer (BTC): tOPAZ‐1. J Clin Oncol. 2022;40:378‐378.

[3] Lamarca A , Palmer DH , Wasan HS , et al. Advanced biliary cancer working, second‐line FOLFOX chemotherapy versus active symptom control for advanced biliary tract cancer (ABC‐06): a phase 3, open‐label, randomised, controlled trial. Lancet Oncol. 2021;22:690‐701. 33798493 10.1016/S1470-2045(21)00027-9 PMC8082275

[4] Banales JM , Marin JJG , Lamarca A , et al. Cholangiocarcinoma 2020: the next horizon in mechanisms and management. Nat Rev Gastroenterol Hepatol. 2020;17:557‐588. 32606456 10.1038/s41575-020-0310-z PMC7447603

[5] Lee YR , Chen M , Pandolfi PP . The functions and regulation of the PTEN tumour suppressor: new modes and prospects. Nat Rev Mol Cell Biol. 2018;19:547‐562. 29858604 10.1038/s41580-018-0015-0

[6] Lee D , Do IG , Choi K , et al. The expression of phospho‐AKT1 and phospho‐MTOR is associated with a favorable prognosis independent of PTEN expression in intrahepatic cholangiocarcinomas. Mod Pathol. 2012;25:131‐139. 21874010 10.1038/modpathol.2011.133

[7] Jiang TY , Pan YF , Wan ZH , et al. PTEN status determines chemosensitivity to proteasome inhibition in cholangiocarcinoma. Sci Transl Med. 2020;12(562):eaay0152. 32967970 10.1126/scitranslmed.aay0152

[8] Jiang TY , Cui XW , Zeng TM , et al. PTEN deficiency facilitates gemcitabine efficacy in cancer by modulating the phosphorylation of PP2Ac and DCK. Sci Transl Med. 2023;15:eadd7464. 37437018 10.1126/scitranslmed.add7464

[9] Jiang TY , Feng XF , Fang Z , et al. PTEN deficiency facilitates the therapeutic vulnerability to proteasome inhibitor bortezomib in gallbladder cancer. Cancer Lett. 2021;501:187‐199. 33220333 10.1016/j.canlet.2020.11.016

[10] Jiang TY , Shi YY , Cui XW , et al. PTEN deficiency facilitates exosome secretion and metastasis in cholangiocarcinoma by impairing TFEB‐mediated lysosome biogenesis. Gastroenterology. 2023;164(3):424‐438. 10.1053/j.gastro.2022.11.025 36436593

[11] Denlinger CS , Meropol NJ , Li T , et al. A phase II trial of the proteasome inhibitor bortezomib in patients with advanced biliary tract cancers. Clin Colorectal Cancer. 2014;13:81‐86. 24512954 10.1016/j.clcc.2013.12.005 PMC4189831

[12] Richardson PG , Briemberg H , Jagannath S , et al. Frequency, characteristics, and reversibility of peripheral neuropathy during treatment of advanced multiple myeloma with bortezomib. J Clin Oncol. 2006;24:3113‐3120. 16754936 10.1200/JCO.2005.04.7779

[13] Bingham V , Ong CW , James J , et al. PTEN mRNA detection by chromogenic, RNA in situ technologies: a reliable alternative to PTEN immunohistochemistry. Hum Pathol. 2016;47:95‐103. 26518664 10.1016/j.humpath.2015.09.009

[14] Abou‐Alfa GK , Macarulla T , Javle MM , et al. Ivosidenib in IDH1‐mutant, chemotherapy‐refractory cholangiocarcinoma (ClarIDHy): a multicentre, randomised, double‐blind, placebo‐controlled, phase 3 study. Lancet Oncol. 2020;21:796‐807. 32416072 10.1016/S1470-2045(20)30157-1 PMC7523268

[15] Abou‐Alfa GK , Sahai V , Hollebecque A , et al. Pemigatinib for previously treated, locally advanced or metastatic cholangiocarcinoma: a multicentre, open‐label, phase 2 study. Lancet Oncol. 2020;21:671‐684. 32203698 10.1016/S1470-2045(20)30109-1 PMC8461541

[16] Saborowski A , Vogel A , Segatto O . Combination therapies for targeting FGFR2 fusions in cholangiocarcinoma. Trends Cancer. 2022;8:83‐86. 34840108 10.1016/j.trecan.2021.11.001

[17] Huang XW , Shi GM , Zhang T , et al. 53P FGFR2 fusion and/or rearrangement profiling in Chinese patients with intrahepatic cholangiocarcinoma. Ann Oncol. 2021;32:S379.

[18] Javle M , Lowery M , Shroff RT , et al. Phase II study of BGJ398 in patients with FGFR‐altered advanced cholangiocarcinoma. J Clin Oncol. 2018;36:276‐282. 29182496 10.1200/JCO.2017.75.5009 PMC6075847

[19] Sun W , Patel A , Normolle D , et al. A phase 2 trial of regorafenib as a single agent in patients with chemotherapy‐refractory, advanced, and metastatic biliary tract adenocarcinoma. Cancer. 2019;125:902‐909. 30561756 10.1002/cncr.31872 PMC6402964

[20] Villanueva L , Lwin Z , Chung HC , et al. Lenvatinib plus pembrolizumab for patients with previously treated biliary tract cancers in the multicohort phase II LEAP‐005 study. J Clin Oncol. 2021;39:321‐321.

[21] Valle J , Wasan H , Palmer DH , et al. A.B.C.T. investigators, cisplatin plus gemcitabine versus gemcitabine for biliary tract cancer. N Engl J Med. 2010;362:1273‐1281. 20375404 10.1056/NEJMoa0908721

[22] Peng W , Chen JQ , Liu C , et al. Loss of PTEN promotes resistance to t cell‐mediated immunotherapy. Cancer Discov. 2016;6:202‐216. 26645196 10.1158/2159-8290.CD-15-0283 PMC4744499

[23] Zhao J , Chen AX , Gartrell RD , et al. Immune and genomic correlates of response to anti‐PD‐1 immunotherapy in glioblastoma. Nat Med. 2019;25:462‐469. 30742119 10.1038/s41591-019-0349-y PMC6810613

[24] Barroso‐Sousa R , Keenan TE , Pernas S , et al. Tumor mutational burden and PTEN alterations as molecular correlates of response to PD‐1/L1 blockade in metastatic triple‐negative breast cancer. Clin Cancer Res. 2020;26:2565‐2572. 32019858 10.1158/1078-0432.CCR-19-3507 PMC7269810

[25] Rosinol L , Oriol A , Rios R , et al. Bortezomib, lenalidomide, and dexamethasone as induction therapy prior to autologous transplant in multiple myeloma. Blood. 2019;134:1337‐1345. 31484647 10.1182/blood.2019000241 PMC6888142

[26] Wu S , Zheng C , Chen S , et al. Subcutaneous administration of bortezomib in combination with thalidomide and dexamethasone for treatment of newly diagnosed multiple myeloma patients. Biomed Res Int. 2015;2015:927105. 26425561 10.1155/2015/927105 PMC4575715

[27] Bold RJ , Virudachalam S , McConkey DJ . Chemosensitization of pancreatic cancer by inhibition of the 26S proteasome. J Surg Res. 2001;100:11‐17. 11516199 10.1006/jsre.2001.6194

